# Taxonomy and Phylogeny of *Polyporus* Group *Melanopus* (Polyporales, Basidiomycota) from China

**DOI:** 10.1371/journal.pone.0159495

**Published:** 2016-08-03

**Authors:** Jun-Liang Zhou, Lin Zhu, Hong Chen, Bao-Kai Cui

**Affiliations:** Institute of Microbiology, Beijing Forestry University, Beijing 100083, China; University of Szeged, HUNGARY

## Abstract

*Melanopus* is a morphological group of *Polyporus* which contains species with a black cuticle on the stipe. In this article, taxonomic and phylogenetic studies on *Melanopus* group were carried out on the basis of morphological characters and phylogenetic evidence of DNA sequences of multiple loci including the internal transcribed spacer (ITS) regions, the large subunit nuclear ribosomal RNA gene (nLSU), the small subunit nuclear ribosomal RNA gene (nSSU), the small subunit mitochondrial rRNA gene sequences (mtSSU), the translation elongation factor 1-α gene (EF1-α), the largest subunit of RNA polymerase II (RPB1), the second largest subunit of RNA polymerase II (RPB2), and β-tubulin gene sequences (β-tubulin). The phylogenetic result confirmed that the previously so-called *Melanopus* group is not a monophyletic assemblage, and species in this group distribute into two distinct clades: the *Picipes* clade and the *Squamosus* clade. Four new species of *Picipes* are described, and nine new combinations are proposed. A key to species of *Picipes* is provided.

## Introduction

*Melanopus* Pat. was established by Patouillard [1] as a specific genus which including stipitate polypores with black stipes. Then it was accepted as a synonym of *Polyporus* P. Micheli ex Adans. [2]. Núñez and Ryvarden [3] treated genus *Melanopus* as an infrageneric group of *Polyporus*. They defined this group with following characters: basidiocarps coriaceous, tough when dry, context thin, stipe with a black cuticle, skeleto-binding hyphae mostly solid and narrow when mature, basidiospores medium size to large (6–12 × 2–4 μm). Eleven species of *Polyporus* were accepted as members of group *Melanopus* by Núñez and Ryvarden [3]. Among these species, *P*. *mikawai* Lloyd was transferred into *Neofavolus* Sotome & T. Hatt. as *N*. *mikawai* (Lloyd) Sotome & T. Hatt. based on morphological and phylogenetic analyses [4].

Phylogenetically, *P*. *badius* (Pers.) Schwein., *P*. *melanopus* (Pers.) Fr. and *P*. *tubaeformis* (P. Karst.) Ryvarden & Gilb. grouped together and formed a clade with higher supports while others dispersed with lower supports [5,6]. Thus, Krüger et al. [6] indicated that “*Melanopus*” appeared to be a non-monophyletic assemblage of dark-stiped polypores. Sotome et al. [7] revealed that *P*. *badius*, *P*. *dictyopus* Mont. and *P*. *tubaeformis* could cluster together in a single clade with high supports while *P*. *leprieurii* Mont., *P*. *varius* (Pers.) Fr., *P*. *squamosus* (Huds.) Fr. and *Datronia mollis* (Sommerf.) Donk, *D*. *scutellata* (Schwein.) Domański, *Pseudofavolus cucullatus* (Mont.) Pat. gathered into another clade. In the latest study of *Melanopus*, a well supported *Melanopus* clade including eleven species was reported [8].

Recently, when studying the lentinoid and polyporoid fungi, Zmitrovich & Kovalenko [9] described a new genus *Picipes* Zmitr. et Kovalenko typified by *Pi*. *badius* (Pers.) Zmitr. et Kovalenko (= *Polyporus badius*) according to analyses of nLSU, ITS and EF1-α datasets. This genus is characterized by the following features: basidiomata annual, stipitate, morphotype polyporoid; pilei infundibuliform, covered with hard cuticle, without scales, smoke gray to castaneous or deeply brown; stipe covered with brownish to black cuticle; pores small (more than 5 per mm); hyphal system dimitic with uninflated both corioiloid and fibrous skeletals; clamps present or absent; basidiospores cylindrical, smooth, hyaline; on wood of frondose and coniferous trees causing a white rot. *Polyporus badius*, *P*. *melanopus* and *P*. *tubaeformis* were segregated from *Polyporus* and transferred into *Picipes* as *Pi*. *badius*, *Pi*. *melanopus* (Pers.) Zmitr. et Kovalenko and *Pi*. *tubaeformis* (P. Karst.) Zmitr. et Kovalenko [9].

In the current study, species diversity of group *Melanopus* in China was investigated, phylogenetic analysis based on 8 genes (including ITS, nLSU, EF1-α, mtSSU, β-tubulin, RPB1, RPB2 and nSSU) was carried out, and four new species matching the concept of *Picipes* are described and illustrated.

## Materials and Methods

All thirty-five specimens examined in this study are publicly deposited in the herbaria of the Institute of Microbiology, Beijing Forestry University (BJFC, Beijing, China) and the Institute of Applied Ecology, Chinese Academy of Sciences (IFP, Shenyang, China). No permits were required for the described study because Chinese legislations do not forbid the access to studying fungi in Nature Reserves and National Parks. We confirm that the studies involve only fungi samples and these samples do not involve endangered or protected species.

### Morphology

Macro-morphological descriptions were based on field notes. Special color terms followed Petersen [10]. Micro-morphological data were obtained from dried specimens, and observed under a light microscope following methods in Li et al. [11]. Microscopic features and measurements were made from slide preparations stained with 5% KOH solution, 1% Congo Red solution, Cotton Blue and Melzer’s reagent. Basidiospores were measured from sections cut from the tubes. To represent variation in the size of basidiospores, 5% of measurements were excluded from each end of the range, and are given in parentheses. The following abbreviations are used: IKI– = neither amyloid nor dextrinoid, KOH = 5% potassium hydroxide, CB+ = cyanophilous, CB– = acyanophilous, L = mean spore length (arithmetic average of all basidiospores), W = mean spore width (arithmetic average of all basidiospores), Q = variation in the L/W ratios between the specimens studied, Qm = mean Q, n (a/b) = number of basidiospores (a) measured from given number (b) of specimens. Microscopic features, such as basidiospores, basidia, hyphae and cystidioles, were observed and photographed at a magnification of up to ×1000 by Nikon Digital Sight DS-Fi1 microscope (Nikon Corporation, Tokyo, Japan), and quantified by the Image-Pro Plus 6.0 software (Media Cybernetics, Silver Spring, USA).

### Molecular phylogeny

CTAB rapid plant genome extraction kit-DN14 (Aidlab Biotechnologies Co., Ltd., Beijing, China) and FH plant DNA kit II (Demeter Biotech Co., Ltd., Beijing, China) were used to extract total genomic DNA from dried specimens and to perform the polymerase chain reaction (PCR), according to the manufacturer’s instructions with some modifications [12,13]. ITS region was amplified with the primer pair ITS4 and ITS5 [14] while nLSU with LR0R and LR7 [15], EF1-α with EF1-983F and EF1-1567R [16], β-tubulin with Bt-1a and Bt-1b [17], nSSU with PNS1 and NS41 [18], mtSSU with MS1 and MS2 [14], RPB1 with RPB1-Af and RPB1-Cr [19], RPB2 with fRPB2-5F and bRPB2-7.1R [20,21]. The primer RPB1-2.2f (GAGTGTCCGGGGCATTTYGG) [22] sometimes was used as an alternative to RPB1-Af and bRPB2-6F [21] was used as an alternative to fRPB2-5F. The final PCR volume is 50 μl each tube which contains 1.5 μl primer (10 pM), 2 μl DNA extract, 20 μl ddH_2_O and 25 μl 2×EasyTaq PCR Supermix (TransGen Biotech Co., Ltd., Beijing, China). PCRs were performed on S1000^™^ Thermal Cycler (Bio-Rad Laboratories, California, USA).

PCR procedures for mtSSU, ITS, nSSU, β-tubulin and EF1-α were as following: (1) initial denaturation 94°C for 2 min, (2) denaturation at 94°C for 45 s, (3) annealing at 52°C (for mtSSU)/53°C (for ITS, nSSU and β-tubulin)/54°C (for EF1-α) for 45 s, (4) extension at 72°C for 1 min, (5) repeat for 36 cycles of last three steps, (6) final extension at 72°C for 10 min.

PCR procedure for nLSU was as following: (1) initial denaturation 94°C for 5 min, (2) denaturation at 94°C for 1 min, (3) annealing at 50°C for 1min 20 s, (4) extension at 72°C for 1 min 30 s, (5) repeat for 36 cycles of last three steps, (6) final extension at 72°C for 10 min.

For RPB1 and RPB2, the PCR procedure was used as following: initial denaturation 94°C for 2 min, followed by 9 cycles at 94°C for 45 s, 60°C for 45 s (minus 1°C per cycle) and 72°C for 1min 30 s, then followed by 36 cycles at 94°C for 45 s, 53°C for 1 min and 72°C for 1min 30 s, finally with a final extension at 72°C for 10 min.

All PCR products were purified and sequenced in the BGI (Beijing Genomics Institute), China, with the same primers. All newly generated sequences were deposited at GenBank and listed in Table 1. *Trametes conchifer* (Schwein.) Pilát was selected as outgroup.

**Table 1:** Species list of *Polyporus* and related genera used in this study and GenBank entries.

No.	Species	Specimen No.	Country	GenBank accession No.							
				ITS	nLSU	EF1-α	mtSSU	β-tubulin	RPB1	RPB2	nSSU
1	*Favolus acervatus*	Cui 11053	China	KU189774 ^a^	KU189805 ^a^	KU189920 ^a^	KU189956 ^a^	KU189864 ^a^	KU189889 ^a^	KU189994 ^a^	KU189835 ^a^
2	*F*. *emerici*	Cui 10926	China	KU189776 ^a^	KU189807 ^a^	KU189922 ^a^	-	KU189866 ^a^	KU189890 ^a^	KU189995 ^a^	KU189837 ^a^
3	*F*. *roseus*	PEN 33	Malaysia	AB735975	AB368099	-	-	-	-	AB368156	-
4	*F*. *spathulatus*	Dai 13615A	China	KU189775 ^a^	KU189806 ^a^	KU189921 ^a^	KU189957 ^a^	KU189865 ^a^	-	-	KU189836 ^a^
5	*Neofavolus alveolaris*	Dai 11290	China	KU189768 ^a^	KU189799 ^a^	KU189913 ^a^	KU189949 ^a^	KU189859 ^a^	KU189885 ^a^	KU189982 ^a^	KU189828 ^a^
6	*N*. *mikawai*	Cui 11152	China	KU189773 ^a^	KU189804 ^a^	KU189919 ^a^	KU189955 ^a^	KU189863 ^a^	KU189888 ^a^	KU189986 ^a^	KU189834 ^a^
7	*Picipes admirabilis*	Dai 1127	China	KC572001	-	-	-	-	-	-	-
8	*Pi*. *americanus*	JV 0509–149 (T)	USA	KC572002	KC572041	-	-	-	-	-	-
9	*Pi*. *austroandinus*	MR 10701	Argentina	AF516569	-	-	-	-	-	-	-
10	*Pi*. *badius*	Cui 10853	China	KU189780 ^a^	KU189811 ^a^	KU189929 ^a^	-	KU189871 ^a^	KU189894 ^a^	-	KU189844 ^a^
11	*Pi*. *badius*	Cui 11136	China	KU189781 ^a^	KU189812 ^a^	KU189930 ^a^	KU189964 ^a^	KU189872 ^a^	KU189895 ^a^	KU189990 ^a^	KU189845 ^a^
12	*Pi*. *badius*	Cui 10501	China	KC572015	KC572053	KU189927 ^a^	KU189962 ^a^	KU189869 ^a^	-	KU189989 ^a^	KU189842 ^a^
13	*Pi*. *badius*	Cui 10484	China	KC572014	KC572052	KU189928 ^a^	KU189963 ^a^	KU189870 ^a^	KU189893 ^a^	-	KU189843 ^a^
14	*Pi*. *baishanzuensis*	Dai 13418 (T)	China	KU189762 ^a^	KU189793 ^a^	KU189907 ^a^	KU189945 ^a^	KU189855 ^a^	KU189882 ^a^	KU189977 ^a^	KU189823 ^a^
15	*Pi*. *baishanzuensis*	Cui 11395	China	KU189763 ^a^	KU189794 ^a^	KU189908 ^a^	KU189946 ^a^	KU189856 ^a^	-	KU189978 ^a^	KU189824 ^a^
16	*Pi*. *conifericola*	Cui 9950	China	KU189783 ^a^	KU189814 ^a^	KU189934 ^a^	KU189968 ^a^	KU189875 ^a^	KU189897 ^a^	KU189993 ^a^	KU189848 ^a^
17	*Pi*. *conifericola*	Dai 11114 (T)	China	JX473244	KC572061	KU189935 ^a^	KU189969 ^a^	-	-	-	KU189849 ^a^
18	*Pi*. *fraxinicola*	Dai 2494	China	KC572023	KC572062	KU189932 ^a^	KU189966 ^a^	-	-	-	-
19	*Pi*. *melanopus*	TENN 59326	Austria	AF518759	-	-	-	-	-	-	-
20	*Pi*. *melanopus*	MJ 400–93	Czech	KC572027	-	-	-	-	-	-	-
21	*Pi*. *rhizophilus*	Dai 11599	China	KC572028	KC572067	KU189933 ^a^	KU189967 ^a^	KU189874 ^a^	KU189896 ^a^	KU189992 ^a^	KU189847 ^a^
22	*Pi*. *submelanopus*	Dai 13294	China	KU189770 ^a^	KU189801 ^a^	KU189915 ^a^	KU189951 ^a^	KU189860 ^a^	KU189886 ^a^	KU189984 ^a^	KU189830 ^a^
23	*Pi*. *submelanopus*	Dai 13296	China	KU189771 ^a^	KU189802 ^a^	KU189916 ^a^	KU189952 ^a^	KU189861 ^a^	-	-	KU189831 ^a^
24	*Pi*. *subtropicus*	Li 1928	China	KU189758 ^a^	KU189790 ^a^	KU189904 ^a^	KU189942 ^a^	KU189854 ^a^	KU189881 ^a^	KU189976 ^a^	KU189820 ^a^
25	*Pi*. *subtropicus*	Cui 2662 (T)	China	KU189759 ^a^	KU189791 ^a^	KU189905 ^a^	KU189943 ^a^	-	-	-	KU189821 ^a^
26	*Pi*. *subtubaeformis*	Dai 11870 (T)	China	KU189752 ^a^	KU189784 ^a^	KU189899 ^a^	KU189937 ^a^	KU189850 ^a^	KU189876 ^a^	KU189972 ^a^	KU189815 ^a^
27	*Pi*. *subtubaeformis*	Cui 10793	China	KU189753 ^a^	KU189785 ^a^	KU189900 ^a^	KU189938 ^a^	KU189851 ^a^	KU189877 ^a^	KU189973 ^a^	KU189816 ^a^
28	*Pi*. *taibaiensis*	Dai 5746 (T)	China	KX196783 ^a^	KX196784 ^a^	KX196785 ^a^	KX196786 ^a^	-	-	-	KX196787 ^a^
29	*Pi*. *tibeticus*	Cui 12215 (T)	China	KU189755 ^a^	KU189787 ^a^	KU189902 ^a^	KU189940 ^a^	KU189853 ^a^	KU189879 ^a^	KU189975 ^a^	KU189818 ^a^
30	*Pi*. *tibeticus*	Cui 12225	China	KU189756 ^a^	KU189788 ^a^	KU189903 ^a^	KU189941 ^a^	-	KU189880 ^a^	-	KU189819 ^a^
31	*Pi*. *tubaeformis*	Niemela 6855	Finland	KC572036	KC572073	-	-	-	-	-	-
32	*Pi*. *tubaeformis*	JV 0309–1	USA	KC572034	KC572072	-	-	-	-	-	-
33	*Pi*. *virgatus*	CulTENN 11406	Argentina	AF516582	AJ488123						
34	*Pi*. *virgatus*	CulTENN 11219	Argentina	AF516581	AJ488122						
35	*Polyporus arcularius*	Cui 11398	China	KU189766 ^a^	KU189797 ^a^	KU189911 ^a^	KU189947 ^a^	-	KU189884 ^a^	KU189980 ^a^	KU189826 ^a^
36	*P*. *brumalis*	Cui 10750	China	KU189765 ^a^	KU189796 ^a^	KU189910 ^a^	-	KU189857 ^a^	KU189883 ^a^	KU189979 ^a^	KU189825 ^a^
37	*P*. *ciliatus*	Wei 1582	China	KU189767 ^a^	KU189798 ^a^	KU189912 ^a^	KU189948 ^a^	KU189858 ^a^	-	KU189981 ^a^	KU189827 ^a^
38	*P*. *dictyopus*	TENN 59385	Belize	AF516561	AJ487945	-	-	-	-	-	-
39	*P*. *guianensis*	TENN 59093	Argentina	AF516564	AJ487947	-	-	-	-	-	-
40	*P*. *guianensis*	TENN 58404	Venezuela	AF516566	AJ487948	-	-	-	-	-	-
41	*P*. *hapalopus*	Yuan 5809 (T)	China	KC297219	KC297220	KU189918 ^a^	KU189954 ^a^	-	-	-	KU189833 ^a^
42	*P*. *leprieurii*	TENN 58579	Costa Rica	AF516567	-	-	-	-	-	-	-
43	*P*. *squamosus*	Wang 555	China	KU189779 ^a^	KU189810 ^a^	KU189926 ^a^	KU189961 ^a^	-	-	-	KU189841 ^a^
44	*P*. *squamosus*	Cui 10595	China	KU189778 ^a^	KU189809 ^a^	KU189925 ^a^	KU189960 ^a^	KU189868 ^a^	KU189892 ^a^	KU189988 ^a^	KU189840 ^a^
45	*P*. *subvarius*	Yu 2 (T)	China	AB587632	AB587621	KU189924 ^a^	KU189959 ^a^	-	-	-	KU189839 ^a^
46	*P*. *tuberaster*	Dai 12462	China	KU507580 ^a^	KU507582 ^a^	KU507590 ^a^	KU507584 ^a^	KU507588 ^a^	-	-	KU507586 ^a^
47	*P*. *tuberaster*	Dai 11271	China	KU189769 ^a^	KU189800 ^a^	KU189914 ^a^	KU189950 ^a^	-	-	KU189983 ^a^	KU189829 ^a^
48	*P*. *umbellatus*	Pen 13513	China	KU189772 ^a^	KU189803 ^a^	KU189917 ^a^	KU189953 ^a^	KU189862 ^a^	KU189887 ^a^	KU189985 ^a^	KU189832 ^a^
49	*P*. *varius*	Cui 12249	China	KU507581 ^a^	KU507583 ^a^	KU507591 ^a^	KU507585 ^a^	-	KU507589 ^a^	KU507592 ^a^	KU507587 ^a^
50	*P*. *varius*	Dai 13874	China	KU189777 ^a^	KU189808 ^a^	KU189923 ^a^	KU189958 ^a^	KU189867 ^a^	KU189891 ^a^	KU189987 ^a^	KU189838 ^a^
51	*Trametes conchifer*	FP 106793sp	USA	JN164924	JN164797	JN164887	-	-	JN164823	JN164849	-

^a^ indicates accession numbers for newly generated sequences; (T) indicates holotype specimen.

Phylogenetic analyses were done as in Zhao et al. [23–25]. All gene sequences were initially aligned separately using Clustal Omega [26] and then manually adjusted. One thousand partition homogeneity test (PHT) replicates of the combined dataset were tested by PAUP 4.0 beta 10 [27] to determine whether the partitions were homogeneous. The best-fit evolutionary model was selected by hierarchical likelihood ratio tests (hLRT) and Akaike information criterion (AIC) in MrModeltest 2.2 [28] after scoring 24 models of evolution by PAUP 4.0 beta 10. The best maximum likelihood (ML) phylogenetic bootstrap values (ML-BS) obtained from 1000 replicates were performed using RAxmlGUI [29], while the ML topology, maximum parsimony (MP) tree and bootstrap values (MP-BS) were performed using PAUP 4.0 beta 10. Bayesian phylogenetic inference and Bayesian posterior probabilities (BPPs) were performed with MrBayes v3.2 [30]. Four Markov chains were run for 5,000,000 generations until the split deviation frequency value <0.01, and sampled every 100th generation. All trees were viewed in FigTree 1.4.2 (http://tree.bio.ed.ac.uk/software/figtree/).

### Nomenclature Acts

The electronic version of this article in Portable Document Format (PDF) in a work with an ISSN or ISBN will represent a published work according to the International Code of Nomenclature for algae, fungi, and plants, and hence the new names contained in the electronic publication of a PLOS article are effectively published under that Code from the electronic edition alone, so there is no longer any need to provide printed copies.

In addition, new names contained in this work have been submitted to MycoBank from where they will be made available to the Global Names Index. The unique MycoBank number can be resolved and the associated information viewed through any standard web browser by appending the MycoBank number contained in this publication to the prefix http://www.mycobank.org/MB/. The online version of this work is archived and available from the following digital repositories: PubMed Central and LOCKSS.

## Results

**Fig 1:**
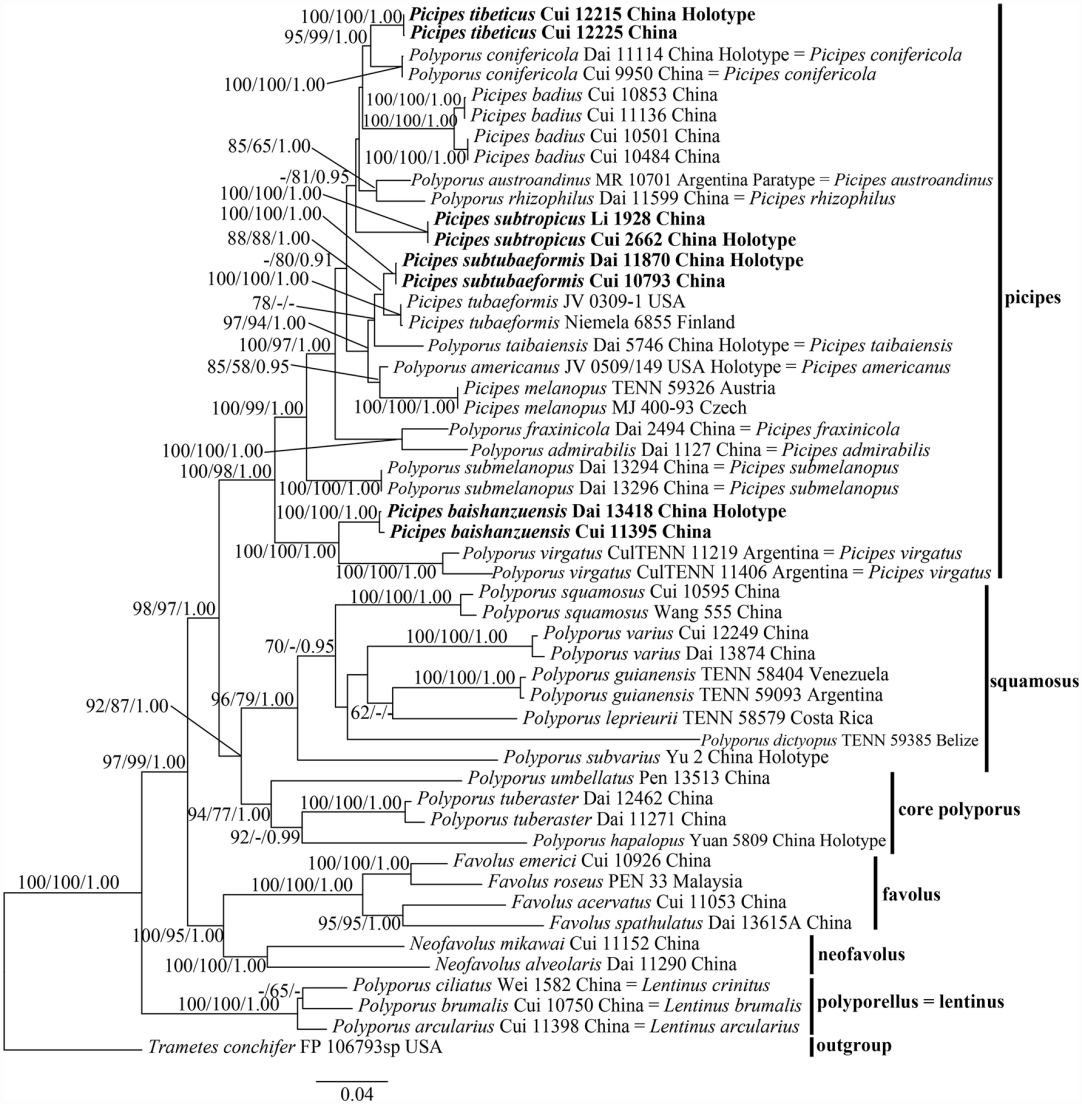
Phylogeny of *Polyporus* and related genera inferred from ITS+nLSU+EF1-α+mtSSU+ β-tubulin+RPB1+ RPB2+nSSU data. Topology is from ML analysis with maximum likelihood bootstrap support values (≥50, former), parsimony bootstrap support values (≥50, middle) and Bayesian posterior probability values (≥0.95, latter). The bold species are new from China.

232 new sequences (Table 1), which including 29 ITS, 29 nLSU, 36 EF1-α, 33 mtSSU, 25 β-tubulin, 22 RPB1, 23 RPB2 and 35 nSSU, were generated for this study. Other 45 related sequences (including 23 ITS, 18 nLSU, 2 RPB2, 1 EF1-α and 1 RPB1) used in phylogenetic analysis were downloaded from GenBank and listed in Table 1. The partition homogeneity test indicated all the eight different DNA sequences display a congruent phylogenetic signal (P value = 0.999). Thus, an 8-gene concatenated dataset resulted in an alignment with 7270 total characters (including 682 ITS + 1364 nLSU + 600 EF1-α + 738 mtSSU + 482 β-tubulin + 1245 RPB1 + 1053 RPB2 + 1106 nSSU nucleotides) was carried out. Among these characters, 4876 of them were constant, 488 variable characters were parsimony-uninformative and 1906 characters were parsimony- informative. In the MP analysis, 63,166,858 rearrangements were tried and two equally most parsimonious trees (length = 8184, CI = 0.481, RI = 0.622, RC = 0.299, HI = 0.519) were retained. While in the ML analysis, 33,295 rearrangements were tried and the best obtained tree scored 46291.85109. ML analysis used the GTR+I+G model and had an identical topology with the Bayesian trees. Topology from ML analysis was presented along with ML-BS (above 50%), MP-BS (above 50%) and BPPs (above 0.95) values (Fig 1, TreeBase submission ID: 18703).

The phylogenetic tree (Fig 1) shows that all sampled species within *Polyporus* group *Melanopus* were divided into two distinct clades:

1. The picipes clade: Besides the three species of *Picipes*, *Pi*. *badius*, *Pi*. *melanopus* and *Pi*. *tubaeformis*, nine *Polyporus* spp. (including *P*. *admirabilis* Peck, *P*. *americanus* Vlasák & Y.C. Dai, *P*. *austroandinus* Rajchenb. & Y.C. Dai, *P*. *conifericola* H.J. Xue & L.W. Zhou, *P*. *fraxinicola* L.W. Zhou & Y.C. Dai, *P*. *rhizophilus* Pat., *P*. *submelanopus* H.J. Xue & L.W. Zhou, *P*. *taibaiensis* Y.C. Dai and *P*. *virgatus* Berk. & M.A. Curtis) and four undescribed *Picipes* species are contained in the well supported picipes clade (100/98/1.00). Among these species, *P*. *rhizophilus*, which was morphologically treated as a member of group *Polyporellus*, is closely related to *P*. *austroandinus* (85/65/1.00), while *P*. *admirabilis* of group *Admirabilis* strongly clusters with *P*. *fraxinicola* (100/100/1.00). The four undescribed species are well supported as new species of *Picipes*.
2. The squamosus clade: *Polyporus squamosus* strongly clusters with *P*. *dictyopus*, *P*. *guianensis* Mont., *P*. *leprieurii*, *P*. *subvarius* C.J. Yu & Y.C. Dai and *P*. *varius* in the squamosus clade (96/79/1.00). Species in this clade usually produce stipes with black cuticle at the lower or entire part, although *P*. *squamosus* was morphologically treated as a member of group *Polyporus*.

The phylogenetic topology also shows four other clades with high supports:

The well supported core polyporus clade (94/77/1.00) contains *P*. *hapalopus* H.J. Xue & L.W. Zhou, *P*. *tuberaster* (Jacq. ex Pers.) Fr. and *P*. *umbellatus* (Pers.) Fr. It shows that *Polyporus* spp. in the core polyporus clade have closer relationships with species in squamosus clade. *Polyporus tuberaster* was selected as the lectotype species of *Polyporus* by Donk [31] and accepted by most succeeding researchers [3,4,7,8,32]. Morphologically, *P*. *tuberaster* was treated as a members of group *Polyporus* along with *P*. *squamosus*, however *P*. *umbellatus* was put into group *Dendropolyporus* [6].

Favolus clade and neofavolus clade are well supported as monophyletic lineages respectively (both 100/100/100 for *Favolus* and *Neofavolus*). These two clades are separately composed of species from genera *Favolus* and *Neofavolus*.

Polyporellus clade, which is composed of *P*. *arcularius* (Batsch) Fr., *P*. *brumalis* (Pers.) Fr. and *P*. *ciliatus* Fr. is well supported as a distinct group phylogenetically separated from other *Polyporus* (100/100/100).

### Taxonomy

***Picipes*** Zmitr. et Kovalenko

Basidiocarps annual, stipitate, of polyporoid morphotype; pilei fan-shaped to circular or infundibuliform, covered with hard cuticle, glabrous; corky to coriaceous when fresh and hard when dry; stipe usually covered with brownish to black cuticle when mature; pores round or angular; hyphal system dimitic with generative and skeleto-binding hyphae, uninflated; generative hyphae with clamp connections or simple septa; skeleto-binding hyphae strongly branched in trama; hyphae in cuticle bearing clamp connections or not, thick-walled with a wide lumen, usually unbranched; basidiospores oblong to cylindrical or fusiform, smooth, hyaline, less than 13 μm long and 5 μm wide; mainly growing on woods and causing a white rot, occasionally on ground or grass roots.

**Fig 2:**
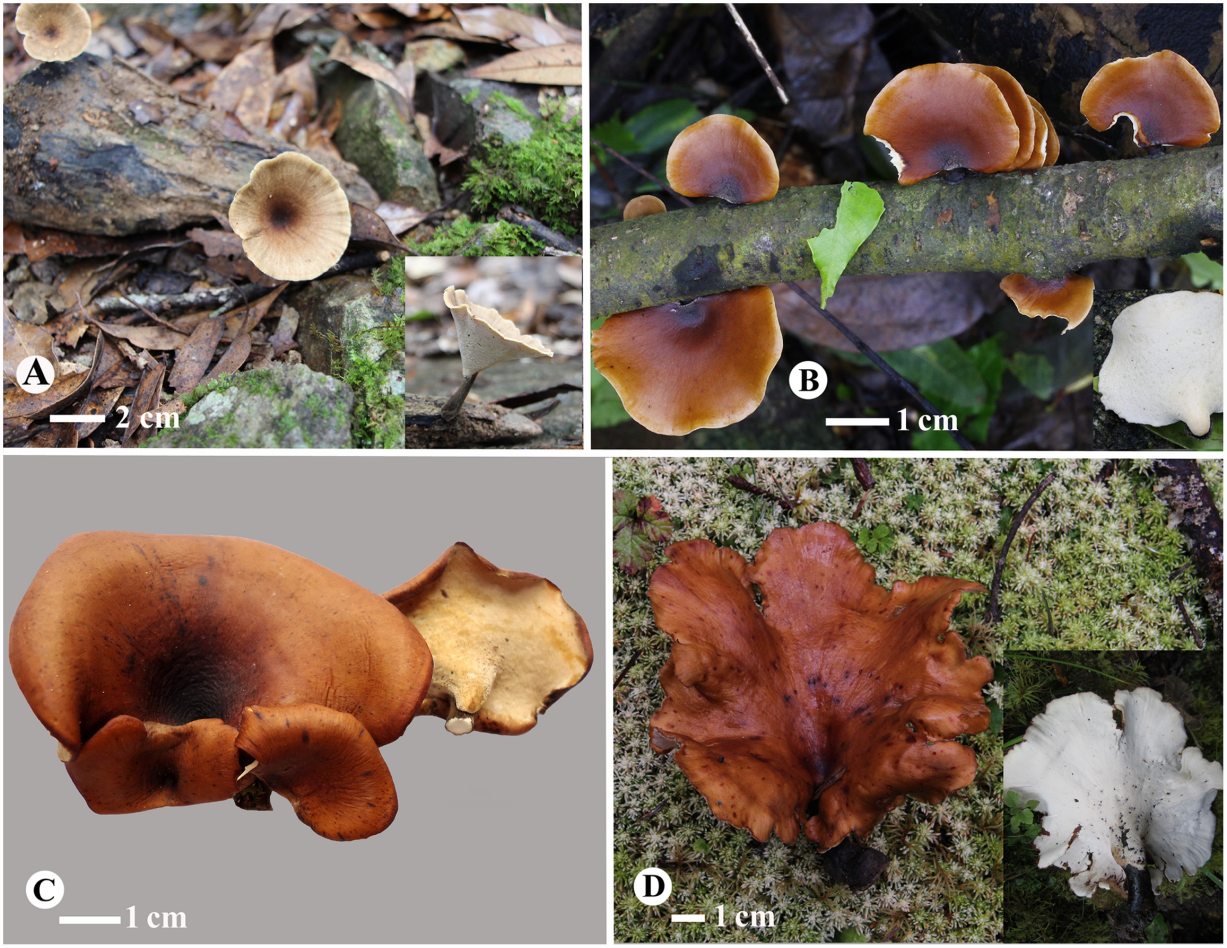
Basidiocarps of the four new *Picipes* species. (A): *Pi*. *baishanzuensis* (Dai 13418); (B): *Pi*. *subtropicus* (Li 1611); (C): *Pi*. *subtubaeformis* (Dai 11870); (D): *Pi*. *tibeticus* (Cui 12215). Bars: A = 2 cm, B, C, D = 1 cm.

**Fig 3:**
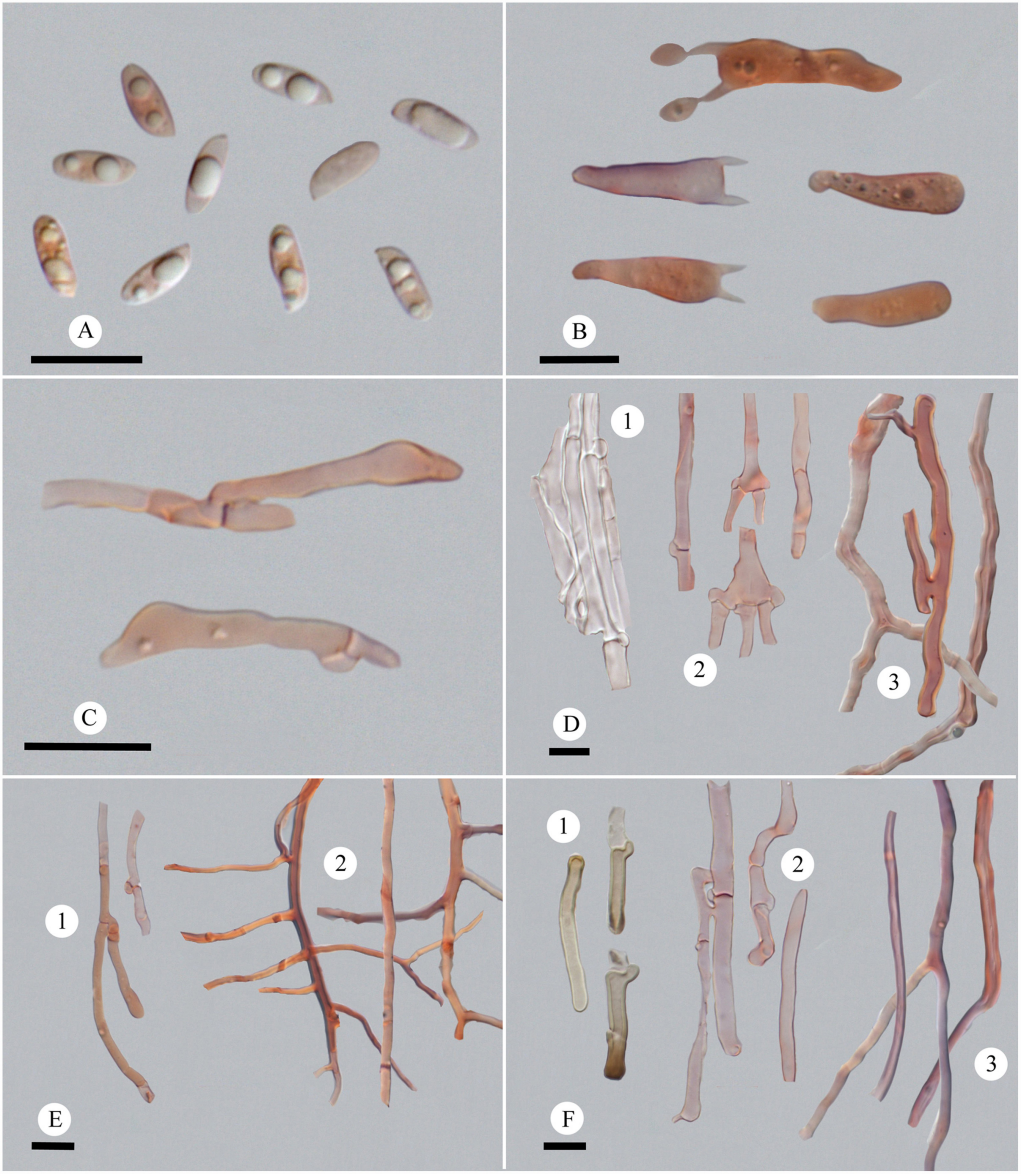
Microscopic structures of *Picipes baishanzuensis*. (A): Basidiospores; (B): Basidia and basidioles; (C): Cystidioles; (D): Hyphae from context, 1 hyphae in cuticle, 2 generative hyphae, 3 skeleto-binding hyphae; (E): Hyphae from trama, 1 generative hyphae, 2 skeleto-binding hyphae; (F): Hyphae from stipe, 1 hyphae in cuticle, 2 generative hyphae, 3 skeleto-binding hyphae. Bars = 10 μm.

1 ***Picipes baishanzuensis* J.L. Zhou & B.K. Cui,** sp. nov. Figs 2A and 3

MycoBank NO.: MB 815517

Basidiocarps annual, centrally stipitate, solitary. Pilei infundibuliform. Pileal surface glabrous, reddish-brown to black in the center and becoming light ivory to pale-brown towards the edge, with radially aligned stripes. Pore surface white; pores round to angular, 3–6 per mm. Stipe black. Hyphal system dimitic; generative hyphae with clamp connections. Basidiospores cylindrical, 6.6–7.9 × 2.5–3.1 μm. On dead angiosperm tree, causing a white-rot.

*Type*: China. Zhejiang Prov., Qingyuan County, Baishanzu Nature Reserve, on dead angiosperm tree, 14 August 2013, Dai 13418 (holotype in BJFC).

*Etymology*: *baishanzuensis* (Lat.): referring to the locality of type specimens in Baishanzu Nature Reserve.

*Fruitbody*: Basidiocarps annual, centrally stipitate, solitary, coriaceous when fresh and tough when dry. Pilei infundibuliform, up to 5.5 cm wide and 2.5 mm thick. Pileal surface glabrous, reddish-brown to black in the center and becoming light ivory to pale-brown towards the edge in young specimens, black upon the whole pileus upon the old ones, with radially aligned stripes; margin straight when fresh and involute upon drying. Pore surface white when fresh, cream to buff upon drying; pores round to angular, 3–6 per mm; dissepiments thin, entire to slightly lacerate. Context white to buff, becoming woody hard upon drying, up to 1 mm thick. Tubes concolorous with pore surface, decurrent on the stipe, less than 1.5 mm thick. Stipe slender, bearing a black cuticle, wrinkled, 2.2 cm long and 5 mm in diam.

*Hyphal structure*: Hyphal system dimitic; generative hyphae bearing clamp connections, colorless, thin-walled; skeleto-binding hyphae colorless, thick-walled, with arboriform branches and tapering ends, IKI–, CB+; tissue unchanged in KOH.

*Context*: Generative hyphae frequent, colorless, thin-walled, frequently branched from clamp connections, 2–5.5 μm in diam., usually inflating at the branching area; skeleto-binding hyphae dominant, colorless, thick-walled with a wide lumen, moderately branched, interwoven, 1.7–6.8 μm in diam. Hyphae in cuticle bearing clamp connections, thin-walled with a wide lumen, with buff inclusion inside, parallel arranged into a palisade, 2.7–6 μm in diam.

*Tubes*: Generative hyphae frequent, usually present near hymenium, colorless, thin-walled, occasionally branched, 2–3.8 μm in diam.; skeleto-binding hyphae dominant, colorless, thick-walled with a wide lumen, frequently with dendroid branching, strongly interwoven, 0.9–3.3 μm in diam. Cystidia absent; cystidioles infrequent, subulate, 16–21 × 3.2–5.3 μm; basidia clavate, with a basal clamp and four sterigmata, 13.4–27 × 4.6–6.5 μm; basidioles in shape similar to basidia, smaller than basidia.

*Stipe*: Generative hyphae frequent, colorless, thin-walled, occasionally branched, 1.9–5.5 μm in diam.; skeleto-binding hyphae colorless, thick-walled with a wide to narrow lumen, moderately branched, interwoven, 1.9–4.3 μm in diam. Hyphae in cuticle bearing clamp connections, thick-walled with a wide lumen, with buff to brown inclusion inside and arranged in a palisade, 3.1–6 μm in diam.

*Basidiospores*: Basidiospores cylindrical, thin-walled, colorless, smooth, usually bearing one to three guttules, IKI–, CB–, (5.8–)6.6–7.9(−8) × (2.4–)2.5–3.1(−3.3) μm, L = 7.04 μm, W = 2.82 μm, Q = 2.14–2.84, Qm = 2.5 (n = 90/3).

*Rot type*: A white rot.

*Additional specimens (paratypes) examined*: China. Zhejiang Prov., Qingyuan County, Baishanzu Nature Reserve, on dead angiosperm tree, 14 September 2012, Cui 11392 (BJFC). Zhejiang Prov., Qingyuan County, Baishanzu Nature Reserve, on dead angiosperm tree, 24 July 2013, Cui 11395 (BJFC).

**Fig 4:**
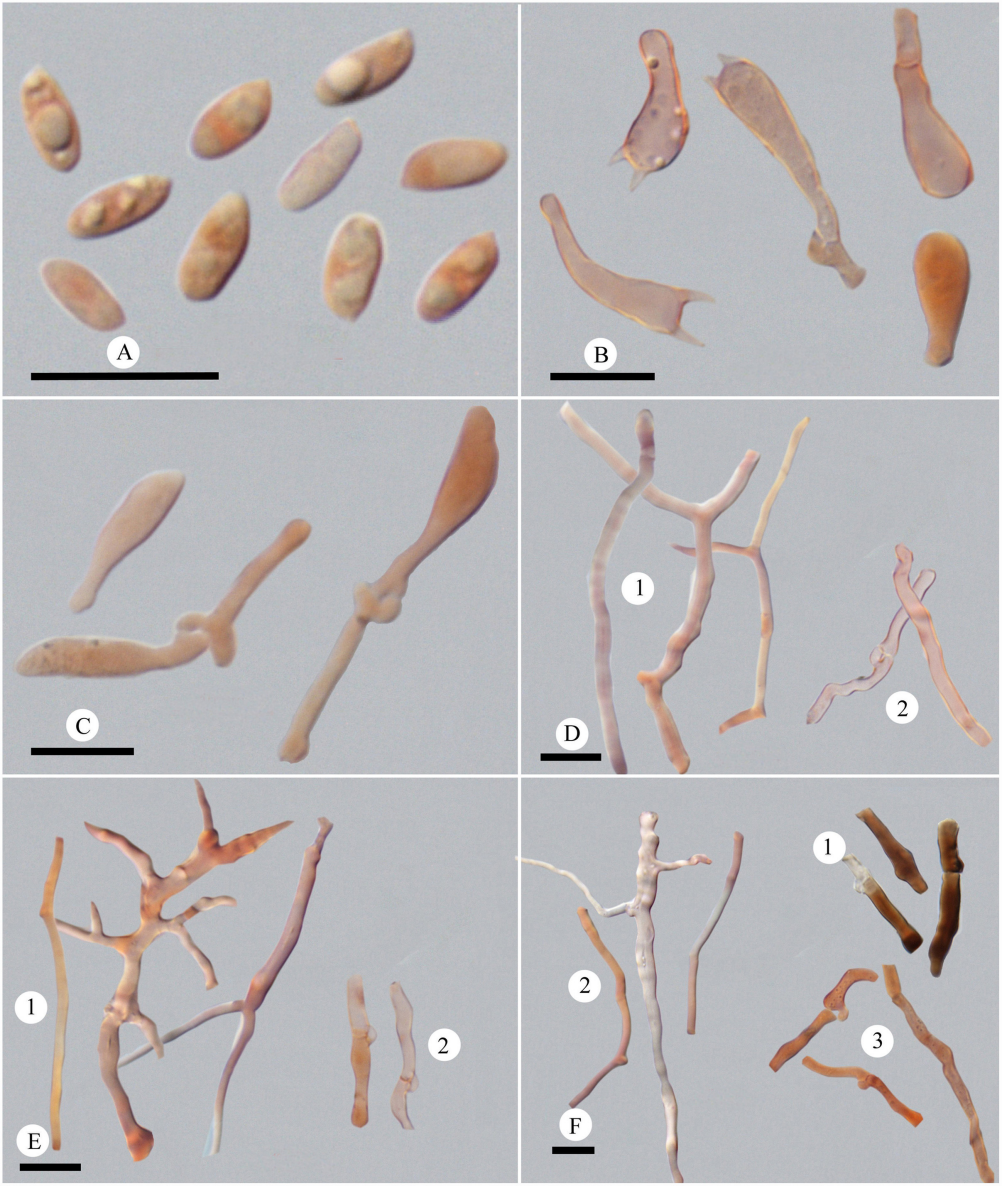
Microscopic structures of *Picipes subtropicus*. (A): Basidiospores; (B): Basidia and basidioles; (C): Cystidioles; (D): Hyphae from context, 1 skeleto-binding hyphae, 2 generative hyphae, 3; (E): Hyphae from trama, 1 skeleto-binding hyphae, 2 generative hyphae; (F): Hyphae from stipe, 1 hyphae in cuticle, 2 skeleto-binding hyphae, 3 generative hyphae. Bars = 10 μm.

2 ***Picipes subtropicus* J.L. Zhou & B.K. Cui,** sp. nov. Figs 2B and 4

MycoBank NO.: MB 815518

Basidiocarps annual, laterally stipitate, gregarious. Pilei fan-shaped to semicircular. Pileal surface glabrous, always black towards the base and becoming reddish-brown to orange-brown towards the edge. Pore surface white; pores angular to subcircular, 8–9 per mm when young and becoming 5–7 per mm when aged. Stipe short or with a flattened base, with a black cuticle. Hyphal system dimitic; generative hyphae with clamp connections. Basidiospores cylindrical, 5.1–6.2 × 2.2–2.7 μm. On fallen angiosperm branch, causing a white-rot.

*Type*: China. Zhejiang Prov., Lin’an, Tianmushan Nature Reserve, on fallen angiosperm branch, 10 October 2005, Cui 2662 (holotype in BJFC).

*Etymology*: *subtropicus* (Lat.): referring to the geographic distribution in subtropical regions.

*Fruitbody*: Basidiocarps annual, laterally stipitate, gregarious, coriaceous when fresh and tough when dry. Pilei fan-shaped to semicircular, up to 4.8 cm wide and 2.5 mm thick. Pileal surface glabrous, black towards the base and becoming reddish-brown to orange-brown towards the edge when fresh, frequently becoming black to chestnut upon the whole pileus, sometimes brown-beige or pastel-yellow towards the edge when dry; margin straight when fresh and straight or slightly involute upon drying. Pore surface white when fresh, white to brown-beige when dry; pores angular to subcircular, 8–9 per mm when young and becoming 5–7 per mm when aged; dissepiments thin, entire to slightly lacerate. Context white to buff and woody hard upon drying, up to 2 mm thick. Tubes white when fresh and white to brown-beige upon drying, less than 1 mm thick, decurrent on one side of the stipe. Stipe very short or forming a flattened base, bearing a black cuticle, up to 5 mm long and 5 mm in diam.

*Hyphal structure*: Hyphal system dimitic; generative hyphae bearing clamp connections, colorless, thin-walled; skeleto-binding hyphae colorless, thick-walled, with arboriform branches and tapering ends, IKI–, CB+; tissue unchanged in KOH.

*Context*: Generative hyphae infrequent, colorless, thin-walled, rarely branched, 1.9–4.7 μm in diam.; skeleto-binding hyphae dominant, colorless, thick-walled with a wide to narrow lumen, moderately branched, interwoven, 1.7–4.4 μm in diam. Hyphae in cuticle bearing clamp connections, thin-walled with a wide lumen, with buff to yellowish-brown inclusion inside, parallel arranged into a palisade, 1.6–3.2 μm in diam.

*Tubes*: Generative hyphae frequent, usually present near hymenium, colorless, thin-walled, 1.5–3.6 μm in diam.; skeleto-binding hyphae dominant, colorless, thick-walled with a wide to narrow lumen, frequently with dendroid branching, strongly interwoven, 1.2–4.2 μm in diam. Cystidia absent; cystidioles frequent, subulate, 14.5–22.8 × 3.2–5.1 μm; basidia clavate, with a basal clamp and four sterigmata, 12.5–27 × 4.8–6.4 μm; basidioles in shape similar to basidia, smaller than basidia.

*Stipe*: Generative hyphae infrequent, colorless, thin-walled, 2–4.5 μm in diam.; skeleto- binding hyphae colorless, thick-walled with a narrow lumen to solid, moderately branched, interwoven, 0.9–5.3 μm in diam. Hyphae in cuticle bearing clamp connections, thick-walled, with a narrow lumen, with dark brown inclusion inside and arranged in a palisade, 2.5–5.6 μm in diam.

*Basidiospores*: Basidiospores cylindrical, thin-walled, colorless, smooth, occasionally bearing one or two guttules, IKI–, CB–, (4.7–)5.1–6.2(–6.6) × 2.2–2.7(−2.9) μm, L = 5.6 μm, W = 2.5 μm, Q = 1.92–2.96, Qm = 2.24 (n = 120/4).

*Rot type*. A white rot.

*Additional specimens (paratypes) examined*: China. Zhejiang Prov., Qingyuan County, Baishanzu Nature Reserve, on fallen angiosperm branch, 14 September 2013, Cui 11393 (BJFC). Guangdong Prov., Fengkai County, Heishiding Nature Reserve, on fallen angiosperm branch, 3 April 2014, Li 1611 (BJFC). Guangdong Prov., Fengkai County, Heishiding Nature Reserve, on fallen angiosperm branch, 3 April 2014, Li 1928 (BJFC).

**Fig 5:**
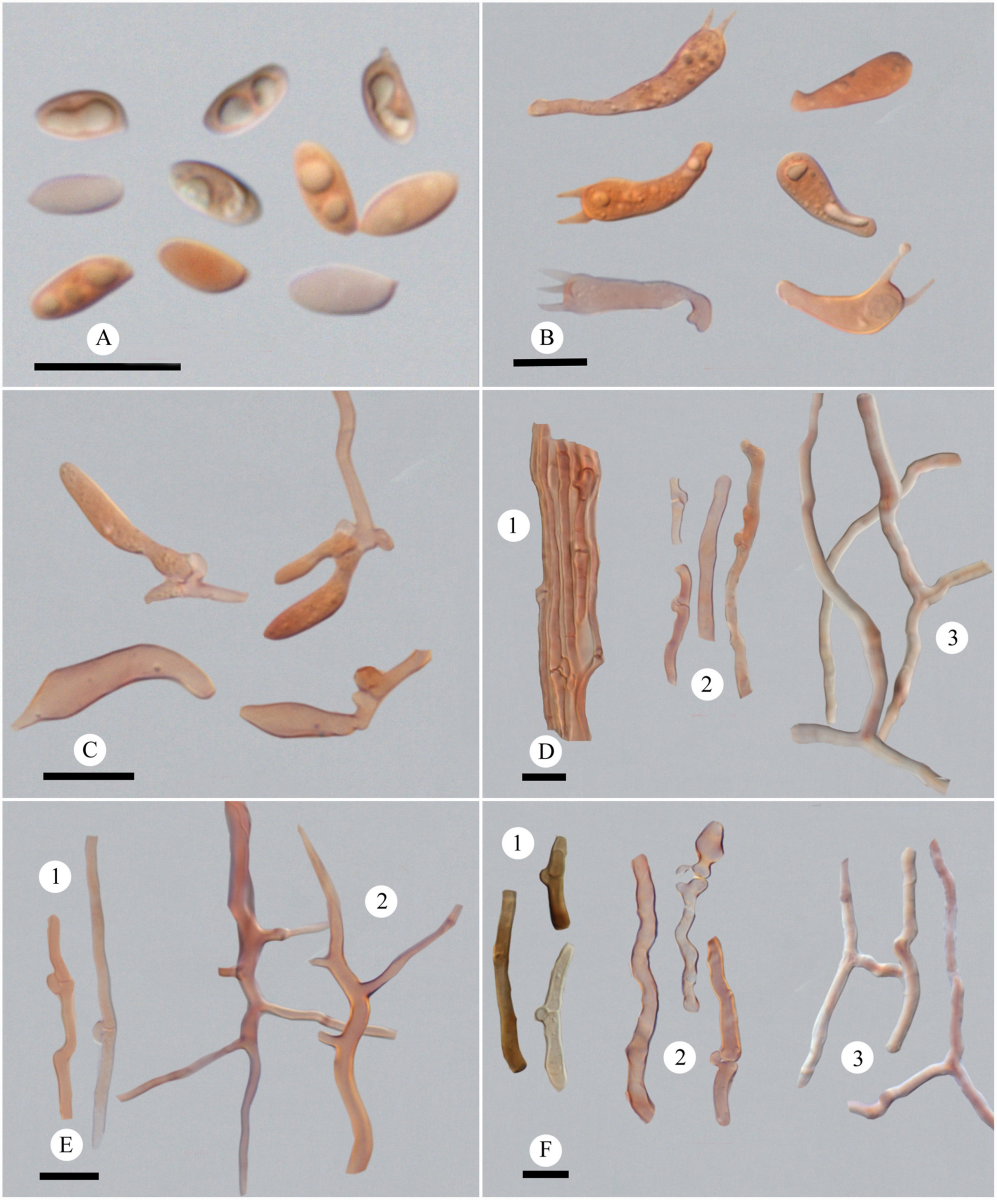
Microscopic structures of *Picipes subtubaeformis*. (A): Basidiospores; (B): Basidia and basidioles; (C): Cystidioles; (D): Hyphae from context, 1 Hyphae in cuticle, 2 generative hyphae, 3 skeleto-binding hyphae; (E): Hyphae from trama, 1 generative hyphae, 2 skeleto-binding hyphae; (F): Hyphae from stipe, 1 hyphae in cuticle, 2 generative hyphae, 3 skeleto-binding hyphae. Bars = 10 μm.

3 ***Picipes subtubaeformis* J.L. Zhou & B.K. Cui,** sp. nov. Figs 2C and 5

MycoBank NO.: MB 815519

Basidiocarps annual, centrally to laterally stipitate, solitary. Pilei irregularly semicircular or elliptical, with shallow central depression. Pileal surface reddish-brown to chestnut in the center or towards the stipe and changing to signal-orange to clay-brown towards the edge. Pore surface buff to festucine, shining; pores angular, 4–6 per mm. Stipe with a terra-brown to black cuticle. Hyphal system dimitic; generative hyphae with clamp connections. Basidiospores oblong to cylindrical, 5.7–6.8 × 2.7–3.1 μm. On angiosperm wood, causing a white-rot.

*Type*: China. Anhui Prov., Huangshan, Huangshan Mountain, on fallen angiosperm branch, 20 October 2010, Dai 11870 (holotype in BJFC).

*Etymology*: *subtubaeformis* (Lat.): referring to the morphological similarity to *Picipes tubaeformis*.

*Fruitbody*: Basidiocarps annual, centrally to laterally stipitate, solitary, coriaceous when fresh and tough when dry. Pilei irregularly semicircular or ellipsoidal, with shallow central depression, up to 7.8 cm wide and 2 mm thick. Pileal surface glabrous, reddish-brown to chestnut in the center or towards the stipe and changing to signal-orange to clay-brown towards the edge when dry in young specimens, becoming reddish-brown to chestnut upon whole surface in mature ones, with radially aligned stripes; margin involute upon drying. Pore surface buff to festucine when dry, shining; pores round to angular, 4–6 per mm; dissepiments thin, entire to lacerate. Context white to buff, becoming woody hard upon drying, up to 1 mm thick. Tubes concolorous with pore surface, less than 1.5 mm thick, sometimes decurrent on one side of stipe. Stipe bearing a terra-brown to black cuticle, up to 1.2 cm long and 3.5 mm in diam.

*Hyphal structure*: Hyphal system dimitic; generative hyphae bearing clamp connections, colorless, thin-walled; skeleto-binding hyphae colorless, thick-walled, with arboriform branches and tapering ends, IKI–, CB+; tissue unchanged in KOH.

*Context*: Generative hyphae infrequent, colorless, thin-walled, frequently branched, 1.6–4.3 μm in diam.; skeleto-binding hyphae dominant, colorless, thick-walled with a narrow lumen to solid, moderately branched, interwoven, 1.4–4.8 μm in diam. Hyphae in cuticle bearing clamp connections, thick-walled with a wide lumen, with buff inclusion inside, parallel arranged into a palisade, 1–3.5 μm in diam.

*Tubes*: Generative hyphae frequent, usually present near hymenium, colorless, thin-walled, 1.5–3.4 μm in diam.; skeleto-binding hyphae dominant, colorless, thick-walled with a wide to narrow lumen, frequently with dendroid branching, strongly interwoven, 1.2–3.6 μm in diam. Cystidia absent; cystidioles frequent, subulate, 16.7–25 × 3.5–5.5 μm; basidia clavate, with a basal clamp and four sterigmata, 15.7–29 × 5.1–6.2 μm; basidioles in shape similar to basidia, smaller than basidia.

*Stipe*: Generative hyphae frequent, colorless, thin-walled, 1.8–6.5 μm in diam.; skeleto-binding hyphae colorless, thick-walled with a narrow lumen, 1–6.4 μm in diam. Hyphae in cuticle bearing clamp connections, thick-walled with a wide lumen, with brown to dark brown inclusion inside and arranged in a palisade, 1.5–4.3 μm in diam.

*Basidiospores*: Basidiospores oblong to cylindrical, thin-walled, colorless, smooth, usually bearing one or two guttules, (5.3–)5.7–6.8(–7.1) × (2.4–)2.7–3.1(−3.4) μm, L = 6.18 μm, W = 2.91 μm, Q = 1.88–2.5, Qm = 2.13 (n = 60/2).

*Rot type*. A white rot.

*Additional specimen (paratype) examined*: China. Sichuan Prov., Luding County, Hailuogou Forest Park, on dead angiosperm tree, 20 September 2012, Cui 10793 (BJFC).

**Fig 6:**
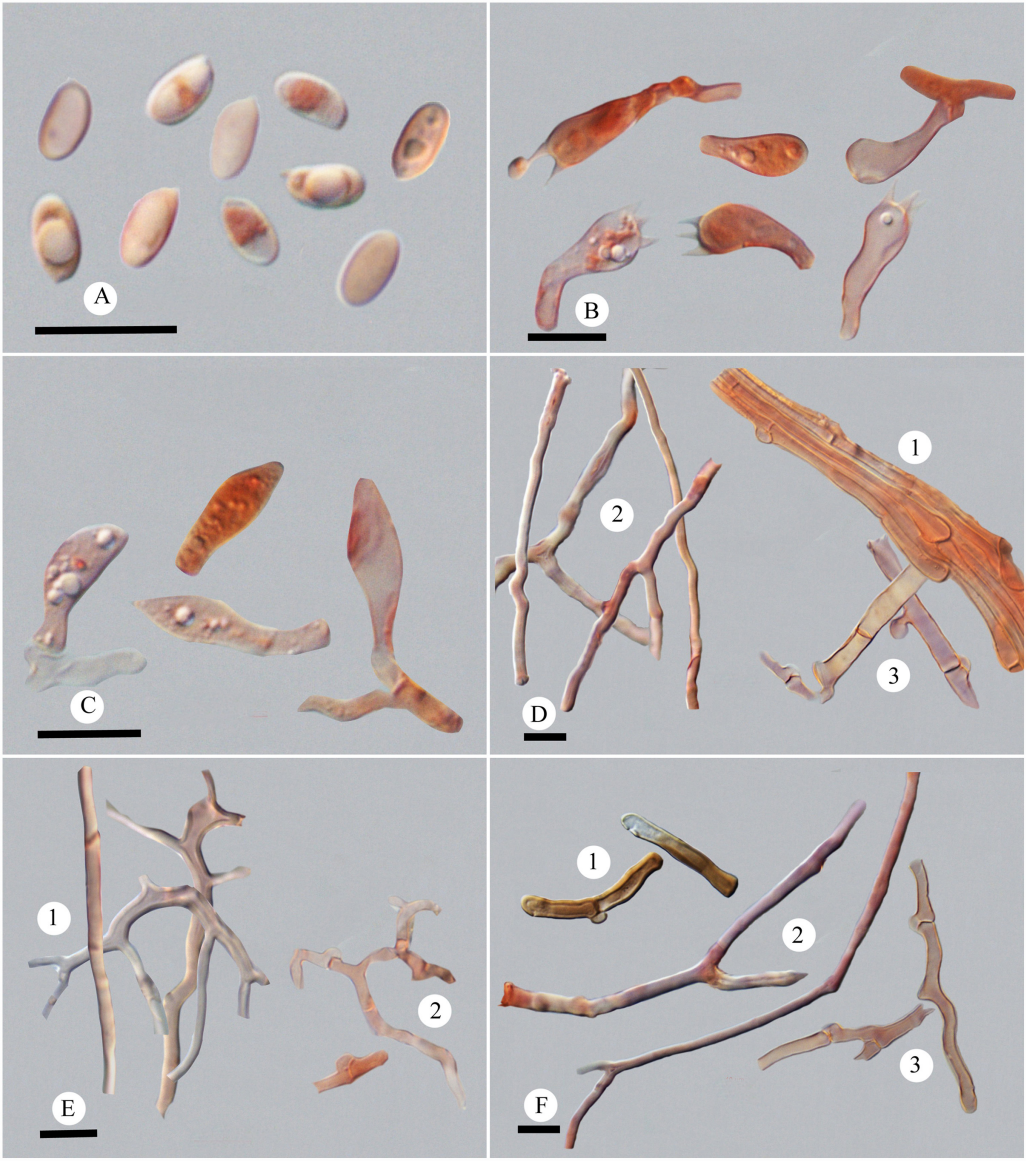
Microscopic structures of *Picipes tibeticus*. (A): Basidiospores; (B): Basidia and basidioles; (C): Cystidioles; (D): Hyphae from context, 1 hyphae in cuticle, 2 skeleto-binding hyphae, 3 generative hyphae; (E): Hyphae from trama, 1 skeleto-binding hyphae, 2 generative hyphae; (F): Hyphae from stipe, 1 hyphae in cuticle, 2 skeleto-binding hyphae, 3 generative hyphae. Bars = 10 μm.

4 ***Picipes tibeticus* J.L. Zhou & B.K. Cui,** sp. nov. Figs 2D and 6

MycoBank NO.: MB 815520

Basidiocarps annual, centrally to laterally stipitate, solitary or scattered. Pilei irregularly fan-shaped or semicircular, usually shallow towards the stipe. Pileal surface orange-brown to brown, more or less radially wrinkled when dry. Pore surface white; pores angular to subcircular, 6–9 per mm. Stipe with a black cuticle. Hyphal system dimitic; generative hyphae with clamp connections. Basidiospores oblong, 5–5.9 × 2.8–3.3 μm. On fallen branch of *Abies* or *Picea*, causing a white-rot.

*Type*: **China**. Xizang Autonomous Region (Tibet), Mêdog County, on fallen branch of *Abies*, 20 September 2014, Cui 12215 (holotype in BJFC).

*Etymology*: *tibeticus* (Lat.): referring to the locality of the type specimens in Tibet.

*Fruitbody*: Basidiocarps annual, centrally to laterally stipitate, solitary or scattered, coriaceous when fresh and tough when dry. Pilei irregular fan-shaped or semicircular, usually shallow towards the stipe, up to 10.5 cm wide and 1 mm thick. Pileal surface glabrous, orange-brown to brown when fresh, becoming orange-brown to reddish-brown or blackish-brown upon drying, more or less radially wrinkled when dry; margin straight or slightly involute when fresh and involute upon drying. Pore surface white when fresh, becoming buff to yellow-orange when dry; pores angular to subcircular, 6–9 per mm; dissepiments thin, entire to slightly lacerate. Context white when fresh, becoming woody hard upon drying, up to 0.5 mm thick. Tubes concolorous with pore surface, less than 0.9 mm thick, decurrent. Stipe bearing a black cuticle, wrinkled, up to 4.5 cm long and 9 mm in diam.

*Hyphal structure*: Hyphal system dimitic; generative hyphae bearing clamp connections, colorless, thin-walled; skeleto-binding hyphae colorless, thick-walled, with arboriform branches and tapering ends; IKI–, CB+; tissue unchanged in KOH.

*Context*: Generative hyphae infrequent, colorless, thin-walled, 2.4–7 μm in diam.; skeleto-binding hyphae dominant, colorless, thick-walled with a narrow lumen to solid, moderately branched, interwoven, 1.6–4.4 μm in diam. Hyphae in cuticle bearing clamp connections, thin-walled with a wide lumen, with buff inclusion inside, parallel arranged into a palisade, 1.6–10 μm in diam.

*Tubes*: Generative hyphae frequent, usually present near hymenium, colorless, thin-walled, occasionally branched, 1.8–3.2 μm in diam.; skeleto-binding hyphae dominant, colorless, thick-walled with a narrow lumen to solid, frequently with dendroid branching, strongly interwoven, 0.9–4.5 μm in diam. Cystidia absent; cystidioles infrequent, subulate, 14.5–21 × 4–5.3 μm; basidia clavate, with a basal clamp and four sterigmata, 15.3–20 × 5.7–6.8 μm; basidioles in shape similar to basidia, smaller than basidia.

*Stipe*: Generative hyphae infrequent, colorless, thin-walled, occasionally branched, 2.2–5.1 μm in diam.; skeleto-binding hyphae colorless, thick-walled with a narrow lumen to subsolid, moderately branched, interwoven, 1.9–6.6 μm in diam. Hyphae in cuticle bearing clamp connections, thick-walled with a wide lumen, with buff to brown inclusion inside and arranged in a palisade, 2.5–5.4 μm in diam.

*Basidiospores*: Basidiospores oblong, thin-walled, colorless, smooth, usually bearing one or two guttules, (4.8–)5–5.9(−6.2) × (2.5–)2.8–3.3(−3.5) μm, L = 5.48 μm, W = 3.01 μm, Q = 1.6–2.07, Qm = 1.82 (n = 90/3).

*Rot type*. A white rot.

*Additional specimens (paratypes) examined*: China. Xizang Autonomous Region (Tibet), Nyingchi County, Lulang, on fallen branch of *Picea*, 24 September 2010, Cui 9651 (BJFC). Xizang Autonomous Region (Tibet), Mêdog County, on fallen branch of *Abies*, 20 September 2014, Cui 12225 (BJFC).

**5 New combinations of *Picipes***

***Picipes admirabilis* (Peck) J.L. Zhou & B.K. Cui, comb. nov.** [MB 817136]

Basionym: *Polyporus admirabilis* Peck, Bulletin of the Torrey Botanical Club 26: 69 (1899). [MB 157762]

≡ *Cerioporus admirabilis* (Peck) Zmitr. et Kovalenko, International Journal of Medicinal Mushrooms 18 (1): 33 (2016). [MB 812034]

***Picipes americanus* (Vlasák & Y.C. Dai) J.L. Zhou & B.K. Cui, comb. nov.** [MB 817137]

Basionym: *Polyporus americanus* Vlasák & Y.C. Dai, Fungal Diversity 64: 136 (2014). [MB 803796]

***Picipes austroandinus* (Rajchenb. & Y.C. Dai) J.L. Zhou & B.K. Cui, comb. nov.** [MB 817138]

Basionym: *Polyporus austroandinus* Rajchenb. & Y.C. Dai, Fungal Diversity 64: 138 (2014). [MB 803797]

***Picipes conifericola* (H.J. Xue & L.W. Zhou) J.L. Zhou & B.K. Cui, comb. nov.** [MB 817139]

Basionym: *Polyporus conifericola* H.J. Xue & L.W. Zhou, Mycological Progress 13(1): 139 (2014). [MB 801216]

***Picipes fraxinicola* (L.W. Zhou & Y.C. Dai) J.L. Zhou & B.K. Cui, comb. nov.** [MB 817140]

Basionym: *Piptoporus fraxineus* Bondartsev & Ljub., Novosti Sistematiki Nizshikh Rastenii 2: 135 (1965). [MB 337052]

≡ *Polyporus fraxinicola* L.W. Zhou & Y.C. Dai, Fungal Diversity 64: 141 (2014). [MB 803799]

***Picipes rhizophilus* (Pat.) J.L. Zhou & B.K. Cui, comb. nov.** [MB 817141]

Basionym: *Polyporus rhizophilus* Pat., Journal de Botanique 8: 219 (1894). [MB 150169]

≡ *Cerioporus rhizophilus* (Pat.) Zmitr. et Kovalenko, International Journal of Medicinal Mushrooms 18 (1): 33 (2016). [MB 812040]

***Picipes submelanopus* (H.J. Xue & L.W. Zhou) J.L. Zhou & B.K. Cui, comb. nov.** [MB 817142]

Basionym: *Polyporus submelanopus* H.J. Xue & L.W. Zhou, Mycotaxon 122: 436 (2013). [MB 800237]

***Picipes taibaiensis* (Y.C. Dai) J.L. Zhou & B.K. Cui, comb. nov.** [MB 817143]

Basionym: *Polyporus rhododendri* Y.C. Dai & H.S. Yuan, Annales Botanici Fennici 46 (1): 58 (2009). [MB 540894]

≡ *Polyporus taibaiensis* Y.C. Dai, Fungal Diversity 64: 142 (2014). [MB 803798]

***Picipes virgatus* (Berk. & M.A. Curtis,) J.L. Zhou & B.K. Cui comb. nov.** [MB 817144]

Basionym: *Polyporus virgatus* Berk. & M.A. Curtis, Botanical Journal of the Linnean Society 10: 304 (1869). [MB 202513]

≡ *Leucoporus virgatus* (Berk. & M.A. Curtis) Pat., Énumération Méthodique des Champignons Recueillis à la Guadeloupe et à la Martinique: 25 (1903). [MB 102236]

## Discussion

*Picipes baishanzuensis* was collected from subtropical area of China. It is characterized by its radially striped infundibuliform pileus with a slender black stipe. In phylogenetic analysis (Fig 1), it strongly clustered with *Pi*. *virgatus* (100/100/1.00). Morphologically, *Pi*. *virgatus* and *Pi*. *baishanzuensis* share infundibuliform pileus, similar pore size, decurrent tubes and wrinkled dark stipe; however, the basidiospores of *Pi*. *virgatus* are much larger (9–12.5 × 4–5 μm for *Pi*. *virgatus* and 6.6–7.9 × 2.5–3.1 μm for *Pi*. *baishanzuensis*) [3]. *Polyporus tuberaster* also has depressed pileus and decurrent pores, but its pileus is covered with dark brown flecks, pores (0.5–2 per mm) and basidiospores (12–14.5 × 4.8–6 μm) [33] are much larger than *Pi*. *baishanzuensis*; besides, *P*. *tuberaster* usually grows on the ground, arising from a black underground sclerotium [3,34].

*Picipes subtropicus* was found in subtropical areas of China. It can be identified by a continuous variation in pore size, bright pileal surface color, short black stipe-like base and medium cylindrical basidiospores (5.1–6.2 × 2.2–2.7 μm). In phylogenetic analysis (Fig 1), it did not cluster with any other species in our study set. *Polyporus dictyopus* also has chestnut upper surface when aged, in addition, its pore size and pore surface are similar to *Pi*. *subtropicus*; but *P*. *dictyopus* has longer and thicker stipe (up to 3 cm long and 1 cm thick), larger basidiospores (7–8.5 × 2.5–4 μm) and pantropical distribution [3]. *Picipes badius* share similar basidiocarps and pore size with *P*. *subtropicus*; but it differs in its larger basidiospores (7.5–9.5 × 3–3.5 μm), simple-septate generative hyphae and absence of cystidioles [3]. *Picipes baishanzuensis* was also found in subtropical areas of China, but its infundibuliform pilei, slender stipe and lager basidiospores (6.6–7.9 × 2.5–3.1 μm) are quite different from *Pi*. *subtropicus*.

*Picipes subtubaeformis* was described from temperate zone of China. It can be distinguished by the irregularly semicircular or elliptical pileus, terra-brown to black stipe, and oblong to cylindrical basidiospores (5.7–6.8 × 2.7–3.1 μm). In the phylogenetic analysis (Fig 1), *Pi*. *subtubaeformis* grouped together with *Pi*. *tubaeformis* (88/88/1.00); morphologically, both of them have orange to reddish-brown pileus and dark stipe, but *Pi*. *tubaeformis* differs in its slender stipe and basidiospores (6–7.8 × 2.3–3.2 μm, L = 6.49 μm, W = 2.75 μm) [35]. Both *Pi*. *virgatus* and *Pi*. *subtubaeformis* have reddish-brown or chestnut basidiocarps with centrally to laterally dark stipe, but the former one has both larger pores (3–4 per mm) and basidiospores [3]; moreover, *Pi*. *virgatus* is absence of cystidioles [3]. *Picipes taibaiensis* is another temperate species described from China. It has similar upper pileal surface color with *Pi*. *subtubaeformis*, but the flabelliform or spathulate pileus, larger basidiospores (7.5–10.5 × 3.2–3.8 μm) and fusoid cystidioles make it different from *Pi*. *subtubaeformis* [36].

*Picipes tibeticus* is a special species found from eastern Tibetan Plateau. it can be identified by its reddish-brown to blackish-brown fan-shaped or semicircular basidiocarps, small angular pores (6–9 per mm), oblong basidiospores (5–5.9 × 2.8–3.3 μm) and growth on coniferous trees. Phylogenetically, it grouped together with *Pi*. *conifericola* (95/99/1.00; Fig 1). Morphologically, *Pi*. *conifericola* and *Pi*. *badius* have similar basidiocarps and substrates as *Pi*. *tibeticus*, but the former two have larger basidiospores (6–8 × 2.3–3.1 μm for *Pi*. *conifericola* [8]; 7.5–9 × 3–3.5 μm for *P*. *badius* [3]). *Picipes submelanopus* resembles *Pi*. *tibeticus* in having dark pileal surface, black-stipitate basidiocarps and buff pore surface, but it differs from *Pi*. *tibeticus* in terrestrial habit, larger pores (2–3 per mm) and basidiospores (8–10 × 3–3.9 μm). In addition, *Pi*. *submelanopus* has both simple septate and clamped generative hyphae [37].

*Picipes admirabilis* was was initially collected on wood of apple trees in northeastern United States [38]. Lloyd considered that *Pi*. *admirabilis* is a variety of *P*. *varius* or belongs to group *Melanopus* for its black stipe [39,40]. Núñez and Ryvarden treated *Pi*. *admirabilis*, *P*. *gayanus* Lév. and *P*. *pseudobetulinus* (Murashk. ex Pilát) Thorn, Kotir. & Niemelä as members of *Polyporus* group *Admirabilis* [3]. Among these three species, *P*. *pseudobetulinus* has recently been combined into *Favolus* as *F*. *pseudobetulinus* (Murashk. ex Pilát) Sotome & T. Hatt. [4]. Recently, Zmitrovich & Kovalenko [9] regarded *Pi*. *admirabilis* as a member of genus *Cerioporus* Quél. But according to our phylogenetic analysis, *Pi*. *admirabilis* strongly clusters in the picipes clade (Fig 1). Morphologically, *Pi*. *admirabilis* has a long (up to 8 cm) and pale buff to black stipe, firm-corky basidiocarps and uninflated hyphae [3]. Based on our specimen collected from northeast of China, the balck stem, corky basidiocarps, uninflated hyphae and strongly branched skeleto-binding hyphae in trama are more similar to species of *Picipes*.

*Picipes rhizophilus* was treated as a member of group *Polyporellus*, and it is a special polypore merely grows on the grass roots [3,7,41,42]. But, recently Zmitrovich & Kovalenko [9] transferred *Pi*. *rhizophilus* into *Cerioporus* as *C*. *rhizophilus* (Pat.) Zmitr. et Kovalenko. In our current phylogenetic analysis, *Pi*. *rhizophilus* strongly groups into the picipes clade (Fig 1). According to our collections, *Pi*. *rhizophilus* has dark brown to black stipe, soft-corky basidiocarps, uninflated hyphae, strongly branched skeleto-binding hyphae in trama and cylindrical basidiospores (8.2–10.1 ×3.2–4.1 μm, L = 9.15 μm, W = 3.63 μm, Qm = 2.52). These above-mentioned features fit *Picipes* well, so we consider this species as a member of *Picipes*.

*Picipes* is showed to be a monophyletic group based on the 8-gen-squences data analysis, and sixteen species are included in this clade (Fig 1). Among these species, *Pi*. *badius* was previously put into *Royoporus* A.B. De by De [43] for its simple septa. But our analysis strongly supported *Pi*. *badius* as a member of *Picipes*. Corner [44] considered that *Pi*. *badius*, *P*. *dictyopus* and *Pi*. *melanopus* were conspecific, but our analysis showed that they are three different species. Zmitrovich & Kovalenko [9] considered that *Picipes* only includes the species with small pores (more than 5 per mm), cylindrical basidiospores and lignicolous habit. According to our study with more samples, we find several species with large pores, oblong basidiospores and terrestrial habit are also members of *Picipes*.

*Polyporus umbellatus* is a particular species that merely grows on the ground from a sclerotium close to stumps of hardwoods, and characterized by numerous stipitate pilei arising from a common, strongly branched stipe [3]. It was morphologically treated as a member of *Polyporus* group *Dendropolyporus* [3]. Phylogenetically, *P*. *umbellatus* was reported as a distinctive species that could not cluster with any other species [7,9]. Zmitrovich & Kovalenko [9] transferred *P*. *umbellatus* into genus *Cladomeris* Quél. But in our analysis, *P*. *umbellatus* strongly clusters with *P*. *tuberaster* and *P*. *hapalopus* in the core polyporus clade (Fig 1). Morphologically, basidiomata of the three species are fleshy when fresh and brittle upon drying [3,45]. Besides, all of them usually have inflated skeleton-binding hyphae up to 17 μm wide, generative hyphae dominant to almost monomitic in trama, cylindrical basidiospores and light-colored stipes. Thus, the core polyporus clade is treated as a natural group of *Polyporus*.

Species in the squamosus clade together with several species of *Datronia* Donk were transferred into *Cerioporus* [9]. Zmitrovich & Kovalenko [9] considered that *Cerioporus* spp. have inflated skeleto-binding hyphae, but the skeleto-binding hyphae of *P*. *guianensis* and *P*. *leprieurii* are uninflated and up to 5 μm wide [3]. So we prefer to maintain their previously taxonomic names here.

Previous phylogenetic analyses showed that favolus clade and neofavolus clade did not gather together and favolus clade has closer relationships with *P*. *tuberaster* [4,8]. But our phylogenetic result (Fig 1) shows that *Favolus* spp. closely related to *Neofavolus* spp. rather than other *Polyporus* spp. Seelan et al. [46] estimated that the ancestral state for *Neofavolus* is angular pores. They transferred *Lentinus suavissimus* Fr., a gilled species with sub-poroid lamellae, into genus *Neofavolus*. Zmitrovich & Kovalenko [9] also treated *Lentinus suavissimus* as a member of *Neofavolus* and named it *N*. *suavissimus* (Fr.) Zmitr. et Kovalenko, but this name is illegitimate because of its earlier homonym *N*. *suavissimus* (Fr.) J.S. Seelan.

The relationships of *Lentinus* Fr. and *Polyporus* have been suspected for a long time. Both Pegler [47] and Singer [48] believed that *Lentinus* divides from polypores, and this assumption had been evidenced by Hibbett & Vilgalys [49] and Hibbett & Donoghue [50]. Molecular phylogenetic studies also showed that species of group *Polyporellus* has a much closer relationship with *Lentinus* compared with other *Polyporus* spp. [4,7,9,46,51–56]. Krüger [5] and Krüger & Gargas [57] proposed a Lentinus–Polyporellus clade alliance to unite species of *Lentinus* s. str. and group *Polyporellus* which have the inflated generative hyphae. Seelan et al. [46] considered the ancestral hymenophoral configuration for species of *Lentinus* and *Polyporellus* group is circular pores, with independent transitions to angular pores and lamellae. Zmitrovich [58] combined species in polyporellus clade into *Lentinus* as *L*. *arcularius* (Batsch) Zmitr., *L*. *brumalis* (Pers.) Zmitr., *L*. *crinitus* (L.) Fr. and *L*. *tricholoma* (Mont.) Zmitr. But the last name is illegitimate because of its earlier homonym *L*. *tricholoma* Berk. & Cooke. Then Zmitrovich & Kovalenko [9] renamed *P*. *tricholoma* to *L*. *flexipes* (Fr.) Zmitr. et Kovalenko. In this article, we prefer to treat species in polyporellus clade as members of *Lentinus*.

Our phylogenetic analysis based on multiple gene sequences data of ITS, nLSU, EF1-α, mtSSU, β-tubulin, RPB1, RPB2 and nSSU suggested that species of group *Melanopus* distribute into two different clades: picipes clade and squamosus clade. This conclusion verified the view that *Melanopus* group is not a monophyletic assemblage of dark-stiped *Polyporus* species, and whether having black cuticle on stipe or not, is not a sufficient feature to define the natural *Melanopus* group. In our study, sixteen species including four new species of *Picipes* are recognized. A key to species of *Picipes* is provided.

**Key to species of *Picipes***
1a Growing on woods or ground………………………………………… 2
1b Growing on grass roots…………………………………………………. *Pi*. *rhizophilus*
2a Generative hyphae bearing simple septa……………………………… 3
2b Generative hyphae only with clamps……………………………………….. 4
3a Pores 2–3 per mm, generative hyphae bearing both simple septa and clamp connections, growing on ground or hardwoods, basidiospores 8–10 × 3–3.9 μm……………………………………………….. *Pi*. *submelanopus*
3b Pores 5–6 per mm, generative hyphae only with simple septa, growing on hardwoods, basidiospores 6.5–8 × 3–3.8 μm……………………………………………………………………. *Pi*. *badius*
4a Stipe very short or attach to the substrate with a flattened base…………………………………… 5
4b Stipe usually more than 1 cm long……………………………………………………………………….. 6
5a Pores 8–9 per mm when young and becoming 5–7 per mm when mature, basidiospores 5.1–6.2 × 2.2–2.7 μm……………………………………………………………………………………… *Pi*. *subtropicus*
5b Pores 3–5 per mm, growing on *Rhododendron*, basidiospores 7.5–10.5×3.2–3.8 μm……………………………………………………………………………………………*Pi*. *taibaiensis*
6a Basidiospores more than 9 μm long…………………………… 7
6b Basidiospores less than 9 μm long……………………………. 8
7a Growing on *Austrocedrus* or *Lomatia* woods, pores 4–5 per mm, basidiospores 9–11.5 × 3–3.8 μm………………………………………………………………………………………………………………………………….*Pi*. *austroandinus*
7b Growing on hardwoods, pores 3–4 per mm, basidiospores 9–12.5 × 4–5 μm…………………………………………. *Pi*. *virgatus*
8a Cystidioles present……………………………………………………..9
8b Cystidioles absent……………………………………………………. *Pi*. *fraxinicola*
9a Pores 3–6 per mm…………………………………………….. 10
9b Pores 6–10 per mm………………………………………………. 14
10a Basidiospores usually more than 3 μm wide…………………………………………. 11
10b Basidiospores usually less than 3 μm wide…………………………………………. 12
11a Cystidioles subulate……………………………………….. *Pi*. *admirabilis*
11b Cystidioles fusoid……………………………………….. *Pi*. *melanopus*
12a Pilei not infundibuliform in shape……………………………………. 13
12b Pilei infundibuliform in shape……………………………………… *Pi*. *baishanzuensis*
13a Pilei nearly circular, basidiospores 7–9 × 2.5–3.1 μm…………………………………….. *Pi*. *americanus*
13b Pilei irregularly semicircular or elliptical, basidiospores 5.7–6.8 × 2.7–3.1 μm……………………………….*Pi*. *subtubaeformis*
14a Basidiospores more than 6 μm in length………………………………….. 15
14b Basidiospores 5–5.9 × 2.8–3.3 μm………………………………………….. *Pi*. *tibeticus*
15a Growing on coniferous trees………………………………… *Pi*. *conifericola*
15b Growing on hardwoods………………………………………. *Pi*. *tubaeformis*
